# Comparison of two pediatric flail chest cases

**DOI:** 10.1186/s13049-015-0156-5

**Published:** 2015-09-25

**Authors:** Ryu Yasuda, Hideshi Okada, Kunihiro Shirai, Shozo Yoshida, Soichiro Nagaya, Haruka Ikeshoji, Kodai Suzuki, Yuichiro Kitagawa, Taku Tanaka, Shiho Nakano, Sho Nachi, Hisaaki Kato, Takahiro Yoshida, Keisuke Kumada, Hiroaki Ushikoshi, Izumi Toyoda, Shinji Ogura

**Affiliations:** Department of Emergency and Disaster Medicine, Gifu University Graduate School of Medicine, 1-1 Yanagido, Gifu, 501-1194 Japan

**Keywords:** Pediatric trauma, Flail chest

## Abstract

Flail chest is a rare complication in pediatric patients with blunt chest trauma. There is no general consensus on which treatment is most appropriate for flail chest in pediatric patients, although it has been reported that surgical fixation is associated with beneficial outcomes for flail chest in adults.

The present report described two pediatric cases of flail chest, which was rare in pediatric blunt trauma. In small children, functional residual capacity is smaller, and the thorax is pliable due to high thoracic compliance. Therefore, it is only advisable to select intubation and mechanical ventilation treatment. Likewise, in pediatric flail chest, the available evidence does not suggest that ventilator management protocols should be adopted routinely, and the treatment for pediatric flail chest was not established completely.

There were not huge different between the described patients, including injury severity and ventilation setting. However, one had a relapse of flail chest after extubation and chest taping was required, while the other patient’s condition was stable after decannulation.

As described above, it is difficult to predict a recurrence of flail chest in pediatric patients even if treatment goes well. Therefore, T-piece trial should be considered prior to extubation.

## Introduction

Flail chest has been reported to occur in 2.2–4.4 % of pediatric patients with blunt chest trauma [1, 2]. There are two main approaches to treating flail chest, surgical fixation and conservative management including mechanical ventilation [3]. A previous study found that surgical fixation is associated with good outcomes for flail chest in adults [4]. However, there is no general consensus on which treatment is most appropriate for flail chest in pediatric patients.

## Case 1

A two-and-a-half-year-old male pedestrian struck by a car was transported to our hospital by air ambulance. His height was 86 cm and his body weight was 14 kg. On arrival his Glasgow Coma Scale score was 11 (eye 3; verbal 3; motor 5). Both pupils were 3 mm, and pupillary light reflexes were normal. Physical examination revealed a body temperature of 37.6 °C, respiratory rate of 41 breaths/min, heart rate of 156 beats/min, and blood pressure of 106/47 mmHg. Paradoxical movement of the left thorax was detected.

Laboratory investigations revealed an inflammatory process and muscle deviation enzyme consistent with blunt trauma. The serum creatinine kinase level was elevated at 1229 IU/l. Arterial blood gas analysis on 100 % oxygen revealed pH of 7.28, PaO_2_ of 73.7 mmHg, PaCO_2_ of 49.3 mmHg, HCO_3_^−^ of 22.5 mmol/l, base excess of −3.6 mmol/l, and lactate of 14 mg/dl.

**Fig. 1:**
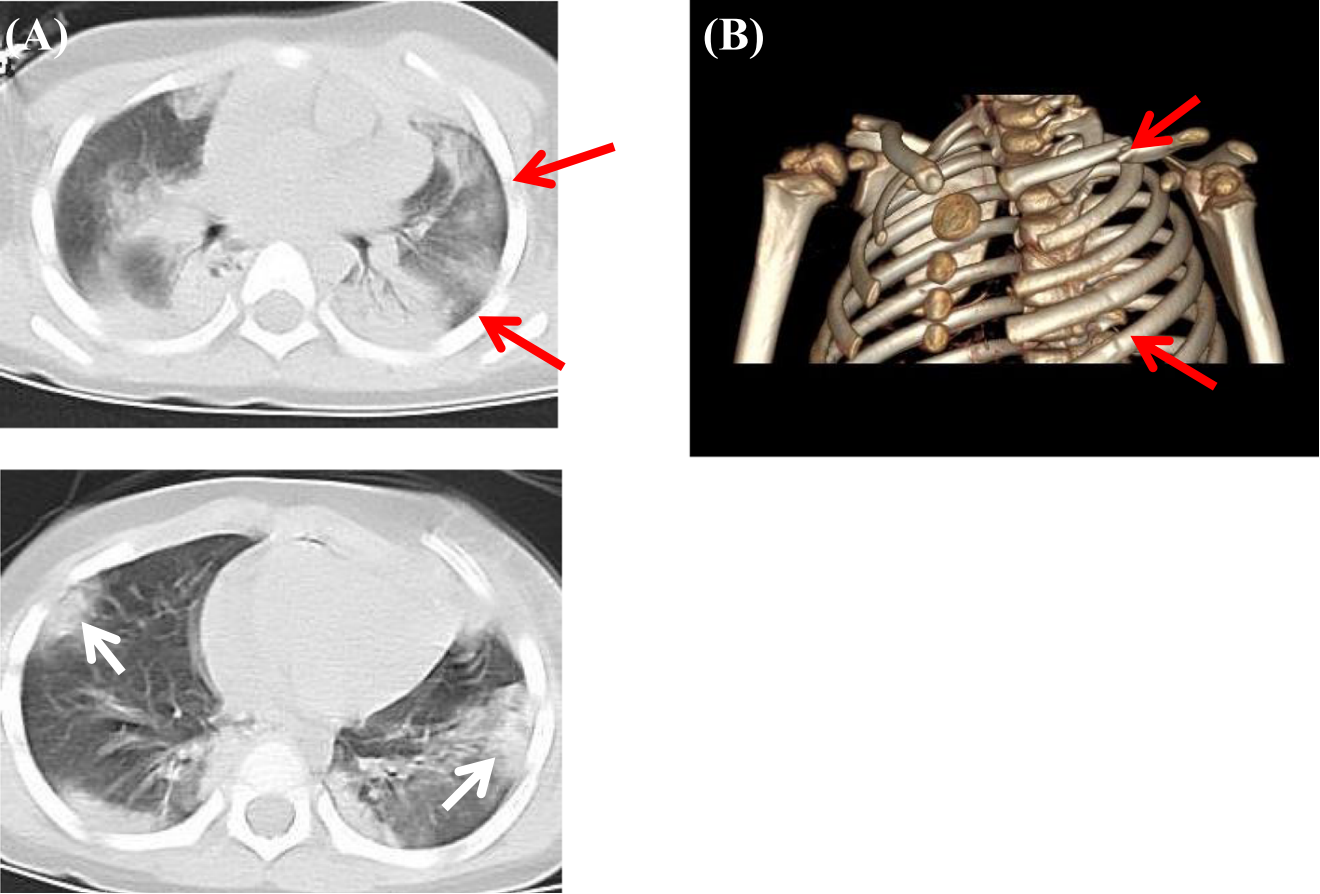
Chest CT of Case 1 on Admission. **a** Two-dimensional axial CT scan showed lung contusions (*white arrows*) and left pneumothorax (*red arrows*). **b** Three-dimensional reconstruction of the CT scan showed fractures of the left clavicle and the third to fifth ribs on the left (*red arrows*)

Chest computed tomography (CT) detected bilateral lung contusions, left pneumothorax and fractures of the left clavicle and left third to fifth ribs (Fig. 1). Based on these findings and paradoxical movement of the left thorax, flail chest was diagnosed. In addition, the patient had liver injury and traumatic subarachnoid hemorrhage. His injury severity score (ISS) was 29.

On admission, the patient was intubated using 4 mm cuffed endotracheal tube. Ventilation mode was synchronized intermittent-mandatory ventilation (SIMV) pressure-control and pressure-triggered ventilation. The initial ventilator settings were: inspiratory time of 0.7 s, respiratory rate of 20 breaths/min, pressure control (PC) 17 cmH_2_O, pressure support (PS) 10 cmH_2_O, positive end expiratory pressure (PEEP) of 9 cmH_2_O and F_I_O2 of 0.4. The PaO_2_/F_I_O_2_ (P/F) ratio, which was 73 on admission, was improved to 375 on hospital day 8. In addition, the lung contusions had improved on chest CT. Then, ventilator mode was continuous positive airway pressure (CPAP) mode, and the patient was requiring minimal ventilatory support. Since paradoxical movement of the left thorax had also resolved, he was extubated on day 9.

Since his condition after extubation was stable, he was transferred to a local hospital for rehabilitation on hospital day 19.

## Case 2

A 1-year-and-8-month-old girl who was run over by a car backing up into a parking spot was transported to our hospital by air ambulance. During transportation, the flail chest was suddenly caused in the air ambulance. In flight, we cannot perform intubation. So, the physician performed manual compression for the flail chest. It was effective and improved the flail chest. Her height was 80 cm and her body weight was 9.3 kg. On arrival her Glasgow Coma Scale score was 9 (eye 2; verbal 2; motor 5). Both pupils were 3 mm, and pupillary light reflexes were normal. Physical examination revealed a body temperature of 37.6 °C, respiratory rate of 50 breaths/min, heart rate of 170 beats/min, and a blood pressure of 120/90 mmHg.

Laboratory investigations showed an inflammatory process and muscle deviation enzyme consistent with blunt trauma. The serum creatinine kinase level was elevated at 3110 IU/l. Arterial blood gas analysis on 100 % oxygen revealed pH of 7.38, PaO_2_ of 90.0 mmHg, PaCO_2_ of 31.9 mmHg, HCO_3_^−^ of 18.7 mmol/l, base excess of −5.0 mmol/l, and lactate of 14 mg/dl.

**Fig. 2:**
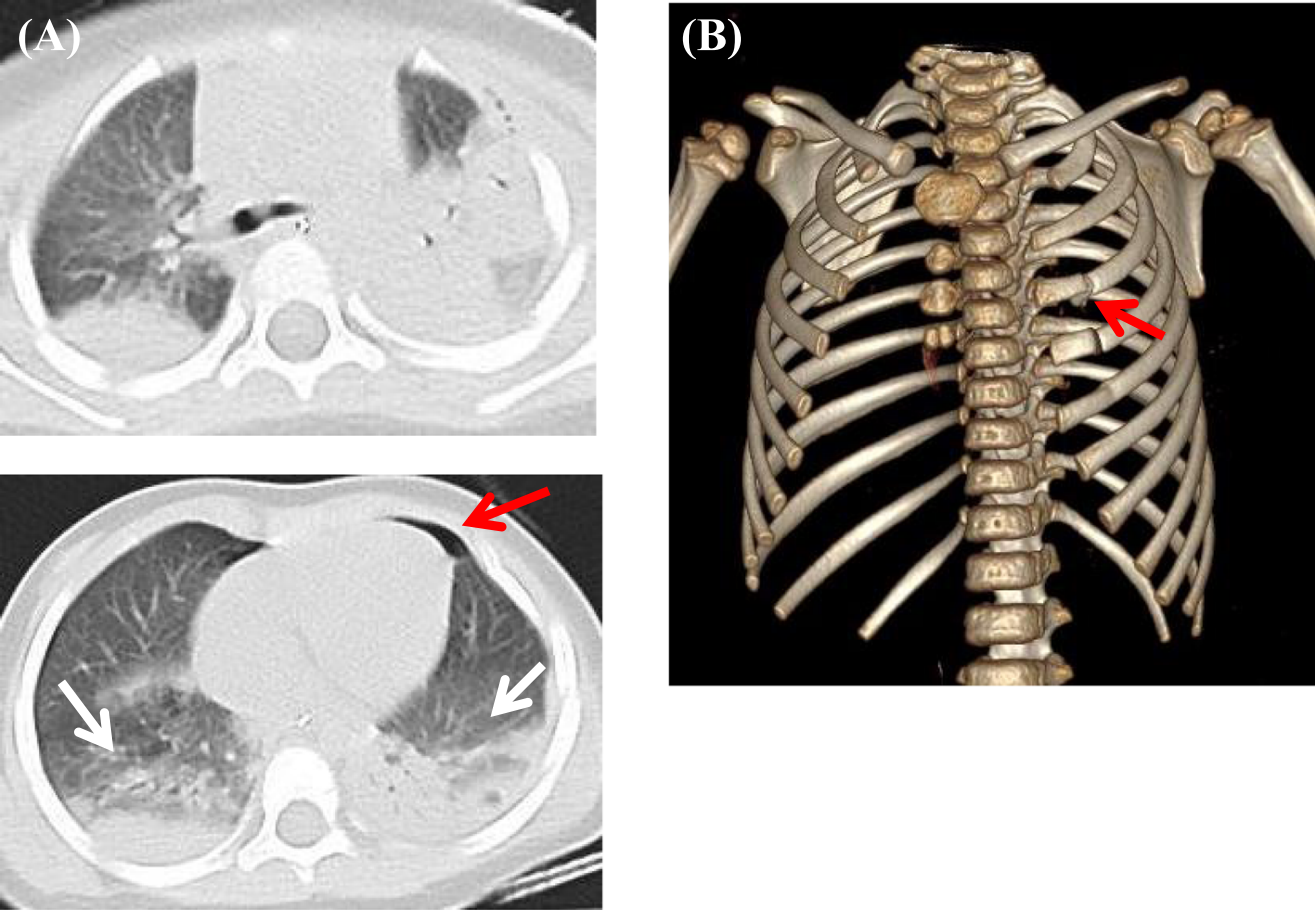
Chest CT of Case 2 on Admission. **a** Two-dimensional axial CT scan showed lung contusions (*white arrows*) and left hemopneumothorax (*red arrows*). **b** Three-dimensional reconstruction of the CT scan showed fractures of the third to fifth ribs on the left (*red arrows*)

Chest CT detected bilateral lung contusions, left hemopneumothorax and fractures of the left third to fifth ribs (Fig. 2). Based on these findings and paradoxical movement of the left thorax, flail chest was diagnosed. The patient also had liver injury, a left femoral diaphyseal fracture, and a left proximal humeral fracture. The ISS was 29.

On the day of admission, the patient was intubated using 4 mm cuffed endotracheal Ventilation mode was synchronized intermittent-mandatory ventilation (SIMV) pressure-control and pressure-triggered ventilation. With an inspiratory time of 0.7 s, respiratory rate of 25 breaths/min, pressure control 13 cmH_2_O, pressure support (PS) 5 cmH_2_O, targeted tidal volume of 70 ml, positive end expiratory pressure (PEEP) of 7 cmH_2_O and F_I_O2 of 0.4.

On day 9, the patient was extubated because paradoxical movement of the left thorax had resolved, lung contusions were improved on chest CT, and the P/F ratio improved from 90 to 300 on minimal spontaneous CPAP ventilation. However, the flail chest relapsed a few hours after extubation. Then, we decided that chest taping was effective if it was short period of time, because the manual compression was effective for this case during transportation and conservative management of mechanical ventilation was performed for 9 days. After all, the presence of a paradoxical segment was disappeared on day 11.

Since her condition was stable, she was transferred to a local hospital for rehabilitation on day 22.

## Discussion

Several recent randomized controlled studies of surgical intervention of flail chest have demonstrated an improvement in the number of ventilator days, intensive care unit and hospital stays, incidence of pneumonia, and respiratory function and hospital costs, as well as faster return to work [4]. Other reports have also described intubation and mechanical ventilation as treatments for flail chest with respiratory failure or multiple severe injuries [5].

Surgical costal fixation is associated with a shorter duration of intubation and hospital stay compared with conservative management including chest taping and mechanical ventilation [3, 6, 7]. In addition, fewer patients treated with surgical costal fixation required tracheotomy and had infectious complications than the conservative therapy group. However, these reports described treatment of flail chest in adults and there is no general consensus on which treatment is more appropriate for flail chest in pediatric patients.

In small children, functional residual capacity is smaller, and the thorax is drawn inside due to high thoracic compliance. It is difficult for small children to stay at rest. Therefore, it is only advisable to select intubation and mechanical ventilation treatment.

Although ventilator management protocols in pediatrics were previously reported [8], in pediatrics flail chest, the available evidence does not suggest that ventilator management protocols should be adopted routinely. Therefore, the treatment for pediatric flail chest was not established completely.

In the case 2, even if flail chest and lung contusion have resolved, it is possible for ventilatory defects due to flail chest to relapse, especially after extubation. In other words, the recurrence is a sudden event and difficult to predict. In fact, although we compared the present cases profiles. (Table 1), there were not obvious different.

**Table 1:** Comparison of two pediatric flail chest

	Case 1	Case 2
Age	2 year-6 month-old	1 year-8 month-old
Body height, weight	86 cm, 13 kg	80 cm, 9.3 kg
Bone fractured area	Left Clavicle Left Third to Fifth Ribs	Left Third to Fifth Ribs
P/F ratio on admission	73 (FiO2: 1.0)	90 (FiO2: 1.0)
Atrial blood gas pH on admission	7.28 (FiO2: 1.0)	7.38 (FiO2: 1.0)
Intubation period	9 Days	9 Days

In conclusion, the present report described two pediatric cases of flail chest, which was rare in pediatric blunt trauma. To manage ventilatory defects secondary to flail chest, conservative medical treatment using a ventilator is the basic treatment. However, it is difficult to predict a recurrence even if treatment goes well. Since chest taping was not universal treatment, the not always work well. Therefore, careful observation is required when extubation, and self-breath test using T-piece before extubation may be enforced.
